# A Simple Matter of Life and Death—The Trials of Postnatal Beta-Cell Mass Regulation

**DOI:** 10.1155/2012/516718

**Published:** 2012-04-22

**Authors:** Elena Tarabra, Stella Pelengaris, Michael Khan

**Affiliations:** School of Life Sciences, Warwick University, Gibbet Hill Road, Coventry CV4 7AL, UK

## Abstract

Pancreatic beta-cells, which secrete the hormone insulin, are the key arbiters of glucose homeostasis. Defective beta-cell numbers and/or function underlie essentially all major forms of diabetes and must be restored if diabetes is to be cured. Thus, the identification of the molecular regulators of beta-cell mass and a better understanding of the processes of beta-cell differentiation and proliferation may provide further insight for the development of new therapeutic targets for diabetes. This review will focus on the principal hormones and nutrients, as well as downstream signalling pathways regulating beta-cell mass in the adult. Furthermore, we will also address more recently appreciated regulators of beta-cell mass, such as microRNAs.

## 1. Introduction

The endocrine cells of the pancreas, located in the islets of Langerhans, are responsible for blood glucose homeostasis, secreting hormones with differing and even opposing effects on blood glucose disposal. Beta-cells, the most numerous islet cells, secrete the hormone insulin which reduces blood glucose levels by increasing peripheral uptake of glucose and by suppressing release of glucose from the liver. Conversely, islet alpha-cells secrete the hormone glucagon which can increase blood glucose levels. Glucagon mainly acts on the liver where it promotes glycogenolysis, releasing glucose from breakdown of glycogen stores and gluconeogenesis. Optimal control of blood glucose levels depends on delicate changes in insulin production and secretion by the pancreatic beta-cells and on their capacity for large increases in secretion after meals, requiring large stores of insulin. It is of critical importance that islets maintain an adequate beta-cell mass in response to various changes.

Recent evidence has revealed that beta-cell replication plays a central role in maintaining adult beta-cell mass [1]. In addition, rates of beta-cell proliferation change dynamically according to metabolic demand throughout life [2]. However, replication of pre-existing beta-cells is not the only available mechanism for generating new beta-cells. In fact, a reasonable body of evidence supports the existence of four other potentially important contributors to adult beta-cell mass regulation: (i) differentiation from stem cells precursors, (ii) transdifferentiation from a non-beta-cell differentiated precursor, (iii) whole islet neogenesis on the plus side and apoptosis on the negative, and (iv) changes in beta-cell size [3, 4]. However, the relative contribution of these processes in maintaining and expanding beta-cell mass is at present not well defined and varies between species [5–7]. During adult life, the beta-cell mass may have to adapt in the face of increased demands due to increases in body mass, pregnancy, or even loss of insulin sensitivity of peripheral tissues. If such compensatory adaptation is inadequate, then glucose homeostasis will be compromised and result in chronically elevated blood glucose, or diabetes [8, 9].

It is well known that beta-cells proliferate extensively during late embryonic development, but the rate of replication slows during postnatal life. During adult life, beta-cell proliferation is detected between 0.5% and 2% [10] gradually declining with age [11]. Remarkably, this low rate of baseline proliferation can be increased significantly in response to pregnancy or obesity and is regarded as an adaptive mechanism in response to increasing systemic insulin demand.

Although important roles of insulin [12] and glucose [13] in beta-cell compensation have been suggested, the mechanism underlying this process is not well understood.

In recent years, various groups have identified microRNAs (miRNAs) small molecules of noncoding RNA that are able to regulate protein expression that contribute to beta-cell dysfunction and diabetes onset [14–18]. However, the role of these miRNAs is not yet fully understood.

Type 2 diabetes (T2D) is characterized by hyperglycaemia resulting from impaired insulin secretion and/or impaired insulin action in peripheral tissues [19]. T2D constitutes one of the greatest pandemics of our time, with 220 million people currently diagnosed [20], and 439 million people expected to be affected by 2030 [21]. Importantly, there is substantial evidence that beta-cell dysfunction plays a major role in the pathology of T2D. For this reason, great efforts are being made in order to develop new therapeutic strategies, such as beta-cell replacement or regenerative medicine.

However, despite progress, most diabetic patients will still die prematurely as a direct result of their disease, its complications, or sometimes even its treatments. In fact, although one may hope that GLP-1 analogues and improved lifestyle may eventually translate into a slowing of T2D progress, clinical trials data have been generally disappointing and confirm that the disease continues to progress [22–25]. To date, no effective treatments for T1D based on slowing or reversing the natural history of the disease exist. Thus we must rely on treatments that can maintain or restore blood glucose levels, and evidences that disease progression can be significantly arrested are scanty. Thus, all T1D and over time most T2D patients will require exogenous insulin (or in rare cases an islet transplant). It is not all negative, and short-term benefits (usually progression from prediabetes to overt diabetes) have been reported in small studies for metformin and acarbose with the common factor likely to be weight loss. The similar success of supervised weight loss programs supports this view. Trials with the PPARg ligand rosiglitazone have also suggested that the progression of T2D may be retarded, but as the drug has been withdrawn, due to an increased risk of cardiovascular disease, this remains interesting rather than clinically useful. In the largest study of T2D to date (UKPDS-UK Prospective Diabetes Study (UKPDS) Group), treatments did not slow progression of patients to additional drugs in order to maintain blood glucose levels at target levels [26].

Furthermore, beta-cells from donor pancreases are in such short supply that transplants can be provided only to a limited number of patients. Ultimately, to cure diabetes, missing beta-cells that must be replaced, and in practical terms this would need to be done from within or a limitless source of beta-like cells must be developed for transplants. One way forward is to “create” beta-cells from alternative cell sources (neogenesis, transdifferentiation, stem cells, etc.). Such an approach requires further knowledge of the mechanisms that regulate pancreatic beta-cell mass.

This review briefly outlines current knowledge of significant factors/nutrients regulating beta-cells mass, and their signal transduction pathways, with greater focus on postnatal regulation and the role of a new class of beta-cell mass regulators: the microRNAs.

## 2. Postnatal Beta-Cell Regulation

**How Do Beta-Cells Maintain Their Normal Mass?**

**Figure 1:**
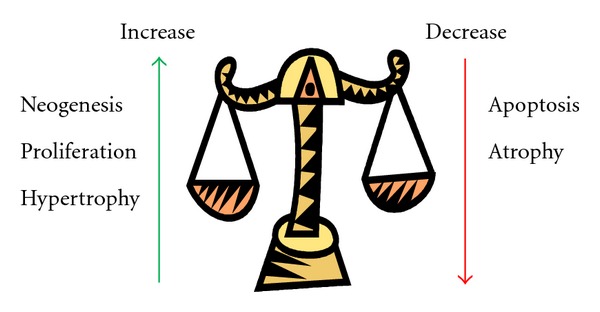
Beta-cell mass equilibrium. Beta-cell proliferation, neogenesis, and hypertrophy (enlarged cell size) increase beta-cell mass, while apoptosis and atrophy (reduced cell size) decrease beta-cell mass.

Beta-cell mass is increased by neogenesis (differentiation from beta-cell precursor cells), proliferation, and cell hypertrophy (increased cell size) and is decreased by beta-cell death, primarily through apoptosis, and beta-cell atrophy (decreased cell size, Figure 1). Several studies have revealed that the primary mechanism by which new beta-cells arise during adulthood is through proliferation of existing beta-cells rather than neogenesis [7, 27]. Thus, organisms born with reduced beta-cell mass have fewer beta-cells available to enter the cell cycle later in life. Under normal circumstances during adulthood, beta-cells are a slowly renewing population, with steady low levels of proliferation and apoptosis. However, beta-cell mass continuously expands over the lifespan of an organism [28], likely due to age-related increases in body weight and insulin resistance. A large number of hormones and nutrients have been shown to affect beta-cell mass, as extensively reviewed by Nielsen et al. [29]. As a result, the number of molecular pathways that have been implicated in beta mass regulation is also large. Many studies have employed animal models, and it is important to note that significant differences between species have been observed [30].

### 2.1. Regulation of Beta-Cell Mass by Hormones, Growth Factors, and other Factors

Beta-cell mass regulation is modulated by various environmental factors and nutrients including glucose, insulin, amino acids, fatty acids, and various other growth factors/hormones, such as IGF-I, IGF-II, glucagon-like peptide-1 (GLP-1) 1, glucagon, gastroinhibitory peptide (GIP), somatostatin (SST), HGF and betacellulin, gastrin, cholecystokinin (CCK), growth hormone (GH), prolactin (PRL), placental lactogen (PL), and leptin, amongst others (Table 1). These growth factors and nutrients can affect a variety of beta-cell functions—suppress/stimulate beta-cell replication, survival, mass expansion, and differentiation through different intracellular pathways discussed later.

**Glucose**

*Glucose* is thought to be the most important determinant of beta-cell mass equilibrium [31–36]. Glucose is not only the pathological hallmark of diabetes, but it is also a potential contributor to further decline in beta-cell mass through what has been termed glucotoxicity. Thus, in the face of evolving demand for insulin, if adaptation (functional or numeric) fails, then blood glucose levels rise, sequentially resulting in IGT and then T2D. Once glucose levels exceed the safe threshold, then further beta-cell death will likely arise [37].

It has been reported that hyperglycaemia for a short time in rats induces beta-cells to enter the cell cycle accompanied by ~50% increase in beta-cell number by neogenesis [33, 38] and suppression of beta-cell apoptosis [33, 36]. In another study using prolonged periods of glucose infusion, an increase of beta-cell mass was also reported, although the origin of these cells has been disputed. While some authors describe proliferation of new beta-cells formed by neogenesis of precursor cells [36], other groups report an increase in beta-cell replication and hypertrophy as well as neogenesis, leading to sustained effects on beta-cell mass even after glucose infusion is stopped [35, 39].

To support the mitogen effect of glucose on beta-cells, Porat and colleagues demonstrated in an animal model an increased replication rate of beta-cells correlated with an increased blood glucose level. Moreover, this effect was mediated by a specific enzyme involved in glucose metabolism (glycolysis) in beta-cells, glucokinase (GCK). In fact, it was demonstrated that GCK has a key role in beta-cell proliferation through membrane depolarization [40].

In addition, glucose has been shown to promote beta-cell survival by suppressing a constitutive apoptotic program *in vitro* [41]. Moreover, it was reported that mild hyperglycaemia can affect beta-cell phenotype [37] with a progressive loss of beta-cell differentiation markers (Pdx-1, BETA2/NeuroD, NKX6.1, and HNF-1*β*) and genes involved in glucose sensing (GLUT-2 and glucokinase) [37, 42]. On the other hand, some genes that are normally suppressed in beta-cells (e.g., c-Myc and its gene target, lactate dehydrogenase-A) are increased. This proapoptotic effect of glucose may also involve altered calcium homeostasis [43], activation of caspases by cytokines, such as IL-1*β* which is secreted by human islets in the presence of high glucose and leads to beta-cell apoptosis [44] or Fas-Fas-Ligand interactions [44], reactive oxygen species [45], and c-Myc [46, 47]. Intriguingly, c-Myc has been shown to trigger apoptosis through all of these pathways in other systems, including in particular the activation of some caspases (caspase 8 and 9), Fas-Fas-Ligand signalling, and reactive oxygen species (reviewed in [48]).

Therefore, paradoxically, it appears that glucose may have both an inhibitory and stimulating effect on beta-cell apoptosis. However, it appears that both the level and duration of hyperglycaemia are crucial in determining the fate of the beta-cell. For example, prolonged hyperglycaemia appears to have a proapoptotic effect, a process referred to as glucose toxicity. Chronic hyperglycemia may thus be injurious to beta-cells and contribute to the development of T2D [49]. Increased beta-cell apoptosis and reduced beta-cell mass have been observed in humans with T2D also, in keeping with the preclinical data in [6, 50–53]. This difference in beta-cell mass between T2D patients and matched controls is particularly evident when those are matched for obesity (obese patients without T2D have increased beta-cell mass compared to lean controls).

**Insulin**

The effect of *insulin* on beta-cell mass has not been completely clarified. Various authors have shown in various *in vivo* studies that insulin alone can stimulate beta-cell mass, whereas other authors have found that it promotes growth only in the presence of hyperglycaemia [34, 36, 54–56].

In a physiological condition, it seems reasonable that during a period of increased insulin demand, insulin itself could stimulate beta-cell growth, creating a positive feedback loop. Further studies have suggested a positive role of insulin in cell regeneration [57, 58].

Genetically altered mice in which the insulin receptor was knocked out exhibited a decreased beta-cell mass in adults and diabetes onset [59]. Insulin resistance, leading to hyperinsulinemia, stimulates an increase in beta-cell mass.

In contrast, as previously described [60, 61], insulin can also impose negative effect on beta-cell mass and/or proliferation. For example, rats with insulinomas resulting in hyperinsulinemia and hypoglycaemia were found to have smaller beta-cells, as well as reduced beta-cell numbers (in the islets without tumour), suggesting that a “sensor” mechanisms exist that can co-ordinate beta-cell mass across islets and possibly even distal grafts [62]. Increased rates of beta-cell apoptosis were evident [60, 61], but interestingly, beta-cell mass regenerated within days of tumour excision [63]. This demonstrates that excess insulin with a negative feedback is able to inhibit beta-cell growth.

These observations indicate that insulin can control beta-cell population dynamics, although the mechanism is still unclear.

**Free Fatty Acids**

Another proposed stimulus for beta-cell mass regulation has been free fatty acids (FFAs). It is well documented that chronic high levels of FFAs, which are often accompanied by obesity, contributes to the pathophysiology of insulin resistance and T2D [64, 65].

It is reported that acute FFA exposure stimulates insulin secretion, while prolonged FFA exposure decreases glucose-stimulated insulin secretion [66, 67] and induces insulin resistance and beta-cell dysfunction in both animal models [68] and humans [69–72].

Animal studies have been conflicting with some arguing that FFAs have a positive trophic effect on beta-cells, whilst others suggest the contrary. However, precise molecular mechanisms linking FFA to beta-cell dysfunction have yet to be fully elucidated.

It was reported that a prolonged *in vitro* exposure of isolated islets or insulin-secreting cell lines to elevated levels of fatty acids is associated with inhibition of glucose-induced insulin secretion [67, 73, 74], impairment of insulin gene expression [75–78], and induction of cell death by apoptosis both *in vitro* [79–88] and *in vivo* studies [80].

Importantly, *in vitro* [76, 88, 89] and *in vivo* [90, 91] studies have provided evidence that lipotoxicity only occurs in the presence of concomitantly elevated glucose levels. A number of studies have shown that fatty acids can induce beta-cell death by apoptosis in the presence of high glucose [88]. *In vitro*, saturated fatty acids induce beta-cell apoptosis [82, 86, 88], whereas unsaturated fatty acids are usually protective [81, 82, 88].

Recently, it was observed by various authors that oxidative stress is implicated in the pathogenesis of beta-cell dysfunction induced by FFAs. It has been suggested that increased reactive oxygen species (ROS) levels are the important trigger for beta-cell dysfunction. Under diabetic conditions, ROS is increased in many tissues and organs and cause various forms of tissue damage in patients with diabetes. It is considered that enhanced ROS generation may act as a link between FFA and beta-cell dysfunction [92].

**Glucagon-Like Peptide-1**

*Glucagon-like peptide-1 (GLP-1)* is produced and secreted in response to fat and glucose from intestinal L-cells, located in the distal ileum and colon.

Preclinical studies reveal potential benefits of GLP-1 receptor agonist treatment in individuals with T2D, which include the promotion of beta-cell proliferation [93] and reduced beta-cell apoptosis [94]. These preclinical results indicate that GLP-1 could be beneficial in treating patients with T2D.

However, the role of endogenous GLP-1 in stimulating beta-cell proliferation is less clear. Data regarding the regenerative role of GLP-1 and related agonists are controversial.

However, there is strong evidence to suggest that GLP-1 receptor activation can protect beta-cells from apoptosis [94–97] and equally convincing evidence of a beta-cell mitogenic effect of GLP-1 [93, 94, 98, 99].

It was reported that exogenous GLP-1 treatment enhances beta-cell replication in normoglycaemic rats [100] and mice [101–105].

However, while there are reports of GLP-1-mediated islet neogenesis [98, 106–109], recent statements by researchers examining the *in vivo *effects of GLP-1 call into question a neogenic response [110–112].

GLP-1 treatment stimulates beta-cell replication in multiple rodent models of obesity-induced diabetes, including both intact [113, 114] and defective leptin signalling [94, 115–117] models. In addition, some authors have demonstrated that exogenous GLP-1 treatment promotes beta-cell replication in models of beta-cell regeneration and obesity-induced diabetes [118, 119].

Moreover, GLP-1 prevents beta-cell death in mouse models of beta-cell loss, in particular, in both unstressed mice [102] and in streptozotocin-induced beta-cell toxicity [95, 120]. In summary, exogenous GLP-1 treatment stimulates beta-cell proliferation in multiple rodent species and models of beta-cell mitogenesis. Reports of human beta-cell proliferation are, however, sparse. Data to support a role for endogenous GLP-1 signalling in beta-cell proliferation are less clear.

**Glucose-Dependent Insulinotropic Polypeptide**

*Glucose-dependent insulinotropic polypeptide (GIP)* is a gut hormone synthesized and secreted from intestinal K-cells, which reside in the duodenum and jejunum.

It was previously reported that if GIP promotes beta-cell proliferation during obesity, then GIP inhibition should reduce beta-cell mass [121].

However, it is unclear whether the primary effect of GIP antagonism is on the adipocyte or the beta-cell. Few studies implicate GIP in *in vivo *beta-cell proliferation [119, 122]. However, one report demonstrated that GIP treatment increases islet size and number in the Leptin ob*/*ob mouse [123]. The latter study did not discriminate between beta-cell proliferation, apoptosis, or islet neogenesis. In a human patient, elevated fasting plasma GIP correlated with islet cell hyperplasia [124]. Despite the limited *in vivo *evidence, *in vitro *experiments demonstrate that GIP enhances beta-cell mitogenesis [125–128].

Furthermore, the role of GIP in the reduction of beta-cell apoptosis *in vivo *is more clearly defined than its role in beta-cell proliferation.

Various studies have also demonstrated that exogenous GIP treatment prevents beta-cell apoptosis during severe obesity [123, 129] and streptozotocin-induced diabetes [120, 129].

**Insulin-Like Growth Factor I and II**

The role of *insulin-like growth factor I (IGF-I) and insulin-like growth factor II (IGF-II)* in beta-cell mass regulation has long been accepted [130–132].

IGF-I has been shown to increase the number of replicating beta-cells in rodent islets by up to 6% of the islet cell population [133–135] and to induce differentiated pancreatic beta-cell growth [133]. However, IGF-I appears only to be effective at inducing beta-cell proliferation under physiologically relevant glucose concentration range: 6 mM to 18 mM [136]. Thus, IGF-I potentiates the mitogenic effect of glucose on beta-cell proliferation.

Interestingly, overexpression of IGF-I in beta-cells is associated with increased beta-cell proliferation, but not mass [137]. In contrast, transgenic mice overexpressing IGF-II exhibit an increase in beta-cell mass due to augmented proliferation [138]. Taken together, there is clear evidence that the IGF molecules are important factors in beta-cell proliferation and mass.

Seemingly paradoxically, beta-cell-specific IGF-I knockout mice showed a delayed onset of T2D induced by a high-fat diet, accompanied by enlarged pancreatic islets, increased insulin mRNA levels, and preserved sensitivity to insulin [139]. This is likely due to secondary increases in insulin and others. Moreover, given the dominant role of circulating IGFs on tissue mass homeostasis in other systems, the significance of this is uncertain. Concerning IGF-II, it has been reported that mice overexpressing this hormone display an increase in beta-cell mass, hyperinsulinemia, and increased glucose-stimulated insulin secretion [140]. These results suggest that IGF-II may have some potential as an islet growth factor.

**Cholecystokinin**

*Cholecystokinin (CCK)* is able to stimulate beta-cell proliferation *in vivo*, as reported by different authors. In fact, various studies have demonstrated that the administration of CCK-8 after streptozotocin treatment in rats expands beta-cell mass via proliferation, in association with fasting plasma insulin increasing, and fasting plasma glucose reduction [141].

At the same time, it was reported that CCK enhances rat beta-cell proliferation *ex vivo*. In fact, neonatal rat beta-cells proliferate in culture after CCK-8 treatment [142]. Similarly, in a rat model, loss of CCK resulted in decreased islet size and reduced beta-cell mass through increased beta-cell death [143]. However, overexpression of *Cck *in mouse and human islets has no effect on replication [143].

**Parathyroid Hormone-Related Protein**

A recent study has demonstrated that acute and systemic administration of the first 36 amino acids of *parathyroid hormone-related protein* (PTHrP (1–36)) in rodents can stimulate beta-cell proliferation and enhance beta-cell mass *in vivo*, without negatively affecting beta-cell function or survival [144]. Previous studies have demonstrated that RIP-PTHrP transgenic mice producing the full-length PTHrP (1–139) in beta-cells show an increase in beta-cell mass, proliferation and islet number, and improvement of glucose homeostasis [145–147]. In addition, *in vitro* studies (in rodent insulinoma cell lines and primary human beta-cells) have shown that PTHrP (1–36) is sufficient to enhance not only proliferation, but also function, increasing insulin mRNA and protein, and augmenting glucose-stimulated insulin secretion [148–150].

However, it is notable that *PTHrP* knockout mice have normal beta-cell mass as compared to controls, suggesting that PTHrP may not be crucial for normal beta-cell mass *in vivo.* However, the explanation of this result should be found on the possibility that the PTHrP was absent in islets but it could be produced in other islet cell types, resulting in a paracrine compensatory mechanism [151].

These findings suggest that PTHrP may provide a possible target for gene therapeutic strategies designed to increase beta-cell mass and function.

**Ghrelin**

*Ghrelin* is a stomach-derived hormone involved in glucose metabolism and has been shown to regulate cellular differentiation and proliferation in various systems. In beta-cells, ghrelin treatment has been shown to increase expression of insulin and PDX1, as well as beta-cell replication in a diabetes model [152]. This suggests a potential to increase beta-cell mass and function.

Moreover, it has been recently reported that both acylated- and unacylated-ghrelin can on the one hand promote cell proliferation while on the other inhibit apoptosis of beta-cells, including human pancreatic islets induced by serum starvation and/or cytokine's stimulation [153, 154].

**Menin**

*Menin* is a tumour suppressor encoded by the *Men1* gene. Mice with heterozygous deletion for *Men1* develop pancreatic hyperplasia [155] that coincides with hyperinsulinemia and hypoglycaemia [156], with an increased islet cell growth through a progressive reduction in expression of some cell growth suppressors, such as p18 and p27 [157]. It was reported that the regulation of p18 and p27 by menin is through histone methylation [158, 159]. Moreover, mutation of *Men1* can induce acceleration in S-phase entry and consequently an enhancement of cell proliferation in pancreatic islets [160]. Recently, it has been reported that menin is also able to increase caspase-8 expression [161] and that caspase-8 is critical for the maintenance of beta-cell mass under physiological conditions [162].

More recently, menin has been demonstrated to play an important role during pregnancy, but this will be discussed below.

**Leptin**

*Leptin* is one of the most important cytokines produced by the *ob* gene and secreted by adipose tissue, and it plays an important role in controlling food intake/satiety and body energy balance (51) through the leptin-receptor (Ob-R).

Ob-R is also present in pancreatic tissue, and it has been proposed that leptin may increase beta-cell proliferation of insulin-secreting cell line [163].

Leptin has also been reported to prevent pancreatic beta-cells from inducible apoptosis, and this may partially account for islet hypertrophy in obese rodents and patients. Leptin may exert its antiapoptotic effects on pancreatic beta-cells. This was associated with a reduction of triglyceride accumulation, an inhibition of nitric oxide production, and a restoration of Bcl-2 expression in the face of fatty acid insult. This may be a possible mechanism for its antiapoptotic effect [164–166].

In the past, it was reported that leptin acted via the JAK/STAT and MAPK pathways and has been shown to increase beta-cell proliferation in cell line studies and foetal pancreatic tissue [167, 168].

However, recent findings indicate that leptin mainly acts through the PI3K-AKT signalling pathway in beta-cell proliferation [169–171]. All these results suggest that leptin has a protective role on beta-cell function. However, the effect of leptin on apoptosis has been conflicting. *In vitro*, prolonged exposure to leptin has been associated with impaired beta-cell function, and increased rates of apoptosis [172].

Furthermore, in contrast to the observations in rodent animal, leptin is likely to have a deleterious effect on human islet function [173]. For instance, it was reported that in isolated islets from mice, leptin was able to increase cell number through the suppression of apoptosis [164] meanwhile in human islets *in vitro* leptin impairs insulin secretion and induces apoptosis of beta-cells in the presence of 20 mM glucose via activation of c-JNK [174].

Overall, leptin is likely to exert diverse effects in the regulation of pancreatic beta-cell function and proliferation. Therefore, further research is still required to clarify its distinct role in various conditions.

**Visfatin**

*Visfatin* is an adipokine secreted by adipose tissue [175], elevated in T2D [176]. This novel compound can mimic the effects of insulin, by binding insulin-receptor, and exerts protective effects on pancreatic beta-cells acting via the PI3K/AKT and MAPK pathways [175]. Through these pathways, visfatin would appear to have the potential to regulate plasma glucose levels, as well as beneficial effects on beta-cell mass.

Overall, visfatin may have a protective effect on pancreatic beta-cell mass, but further research is necessary to clarify its distinct roles.

**Placental Hormones**

These hormones (especially *placental lactogen* and *prolactin*) are principally responsible for the changes in beta-cell mass during pregnancy (reviewed in [177, 178]). These hormones stimulate beta-cell proliferation in isolated islets, and beta-cell mass is reduced 26–42% in receptor deficient mice [179] as described in a later.

**Connexin**

Beta-cell-cell interactions are mediated by *connexins (Cxs)*. These proteins form the gap junction domains of cell membranes. Cxs have been shown to affect cell proliferation and survival in different cell types [180–182]. In particular, it was reported that the Cx36 isoform is the only one expressed in rodent beta-cells [183]. A recent study performed by Klee et al. [184], using Connexins-inducible/knockout transgenic mouse models, reported a key role in beta-cell mass regulation by demonstrating that the native Cx36 does not alter islet size or insulin content, whereas the Cx43 isoform increases both parameters, and Cx32 has a similar effect only when combined with the GH.

**Bone Morphogenetic Proteins**

As recently reported, there are some new mediators that are responsible of proliferation/apoptosis of beta-cells. Bone morphogenetic proteins (BMPs) are growth factors involved in the pancreas mass homeostasis both during embryonic development and adult life [185, 186]. The binding of these molecules, in particular BMP1 and BMP4, to their specific receptors induce, the phosphorylation and nuclear translocation of SMADs. In addition, there were identified different target genes able to regulate different transcription factors (ID1, ID2, ID3, ID4) and proteins involved both in the proliferation and apoptosis processes (including GK, IRS, E2F, SMAD7/9, HES-1). This is the reason why BMPs have a double effect, both proliferative and proapoptotic.

### 2.2. Signalling Pathways in Beta-Cell Mass Homeostasis

An ever expanding literature suggests that the above-described hormonal and nonhormonal factors are able to regulate beta-cell mass by cell cycle regulatory proteins and a range of key signalling pathways. Of particular interest in the light of recent data are mitogenic and apoptotic signalling pathways regulated by the transcription factor c-Myc [46], downstream targets involved in G_1_/S transition [187–195], and various relevant pathways, including PI3K-AKT and MAPK. 

In particular, the PI3K-AKT pathway is an important mediator of beta-cell mass increase [5, 136, 196]. In fact, PI3K-AKT is known to play a major role in preventing c-Myc apoptosis in many cell types [197] and to promote hypertrophy, hyperplasia, and neogenesis [5].

**Phosphatidylinositol 3-Kinase/AKT Pathway**

**Figure 2:**
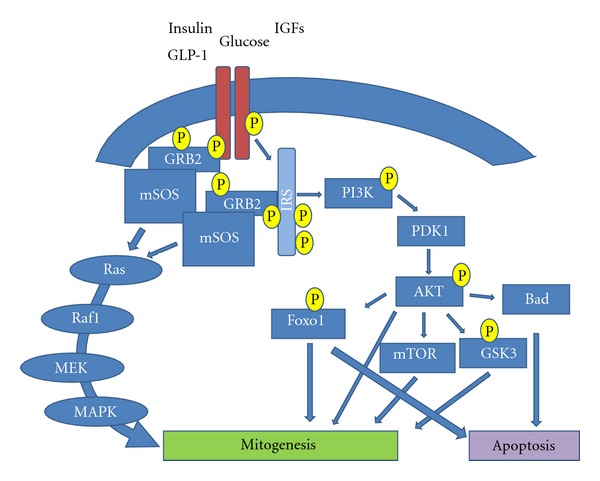
Schematic representation of PI3K/Akt and MAPK signalling. Mechanisms of some growth factors modulate beta-cell replication through proliferation or apoptosis. These factors on one hand stimulate PI3K, AKT, MAPK, and increase beta-cell proliferation. On the other hand, stimulate AKT, FOXO1, and other proapoptotic molecules resulting in induction of apoptosis.

There are many signal transduction pathways involved in the regulation of beta-cell mass, with phosphatidylinositol 3-kinase (PI3K) and AKT signalling being one of the best defined [198]. This pathway is activated by factors, such as glucose, insulin, IGFs, and GLP-1 [136, 199], and is able to regulate beta-cell size, proliferation, and apoptosis. Following ligand-receptor binding, insulin receptor substrate (IRS) is phosphorylated and is able to interact with various downstream targets. The most important target of IRS is PI3K, which in turn can activate PDK1 (via a secondary messenger) [200], resulting in the activation of AKT [200, 201]. AKT plays diverse roles in the beta-cell, including activation of glycogen synthase kinase-3 (GSK3, described later), and phosphorylation and nuclear exclusion of Forkhead transcription factor O1 (FOXO1) [202], ultimately leading to cell growth/replication and suppression of apoptosis [191] (described in Figure 2).

This is further evidenced in *IRS2* ^−/−^ mice, whereby beta-cell proliferation was achieved by ablation of one allele of *FOXO1* [203].

It was reported that PDX1 transcription is regulated positively by forkhead transcription factor A2 (FOXA2) but negatively by FOXO1 [204, 205]. In fact, these 2 forkhead transcription factors share common DNA-binding sites in the *Pdx1* promoter, FOXO1 competes with FOXA2 for the same binding site resulting in the inhibition of *Pdx1* transcription [204]. Moreover, nuclear translocation of FOXO1 excludes PDX1 from the nucleus, or vice versa [204, 206]. In addition, constitutively active nuclear FOXO1 has been shown to inhibit beta-cell proliferation in the face of insulin resistance [207]. This serves as a second mechanism by which FOXO1 inhibits beta-cell proliferation.

Recently, studies of beta-cells exposed to low nutrition revealed an additional beneficial role of FOXO1 activation—beta-cell proliferation without any changes in apoptosis compared to the control group [208]. Moreover, this study revealed that PI3K and MAPK signalling pathways are dampened and that the induction of CyclinD1 expression by activated FOXO1 in low nutrition is responsible for the improved proliferation of beta-cells [208].

Various studies using animal models to evaluate the role of different molecules on the PI3K/AKT pathway and beta-cell mass regulation have been performed.

Both IRS1 and 2 are critical for beta-cell growth and survival (previously mentioned), and they occupy a key position between the insulin receptor and downstream signaling elements. *IRS1* ^−/−^ knockout mice develop severe insulin resistance [209, 210] and compensate for insulin resistance by increasing beta-cell mass [211]. Although *IRS2* ^−/−^ mice are born with only a slightly reduced beta-cell mass, they later develop diabetes due to a marked increase in spontaneous apoptosis and reduced survival of beta-cells [209]. It, therefore, appears that IRS2 plays an important role in maintaining beta-cell homeostasis.

Furthermore, the role of AKT has been studied in beta-cells using genetically altered mice in which AKT is knocked out or overexpressed. AKT knockout mice displayed an increase of blood glycaemia and insulin resistance, although there have been conflicting reports on the effect on compensatory beta-cell growth [212]. Overexpressing AKT in transgenic mice led to a significant increase in beta-cell mass [5, 213]. In addition, AKT has been shown to promote beta-cell survival *in vivo* [214], suggesting an important role for AKT in beta-cell mass regulation.

The role of FoxO1 on signal transduction was further evidenced in different mouse models. It was demonstrated that constitutively active form of FoxO1 protects *β* cells from oxidative-stress-induced damage [197], and using another mouse model, it was demonstrated that deletion of one allele of FoxO1 resulted in a marked increase in the number, but not the size [209].

The role of PDX1 in beta-cell biology has been predominately examined in knockout studies. However, inactivating mutations in one *Pdx-1* allele have yielded conflicting results, although a “critical” level of PDX-1 is thought to be required in order to maintain beta-cell mass that [215, 216].

**GSK3**

Glycogen synthase kinase-3 (GSK3), a constitutively active serine/threonine kinase, was the first substrate shown to be phosphorylated by AKT (8).

Mice overexpressing a constitutively active form of GSK3 in beta-cells display reduced levels of PDX-1 protein [217].* In vitro *studies confirmed the role of GSK3 in regulating PDX-1 protein stability, for example, in MIN6 cells, GSK3 has been shown to phosphorylate PDX-1 leading to its proteasomal degradation. These results highlighted a new mechanism, whereby signalling through the PI3K/AKT/GSK3 pathway regulates beta-cell growth [217]. In addition to the modulation of PDX-1 stability, GSK3 can also regulate beta cell proliferation through phosphorylation and degradation of cell cycle proteins, such as cyclin D1 and/or p27 [218, 219]. Other targets of GSK3 include numerous transcription factors involved in the cell cycle including c-Myc and c-Jun [220]. Therefore, in response to different growth factors, AKT prevents GSK3 phosphorylation of cyclin D, thereby promoting cell cycle progression and subsequent mitogenesis (Figure 2).

**Mitogen-Activated Protein-Kinase (MAPK) Pathway**

IRS can activate the RAF-MEK-ERK pathway (aka the mitogen-activated protein-kinase (MAPK) pathway, Figure 2) which plays a crucial role in development, cellular differentiation, and mitogenesis [221]. Firstly, IRS engages the adaptor molecule growth factor receptor-bound protein-2 (Grb2) via its SH2 domain [222, 223]. In turn, Grb2 binds to the murine Son-Of-Sevenless-1 protein (mSOS), which is able to activate RAS protein [222, 223]. It then, associates with RAF serine kinase [224], which subsequently phosphorylates mitogen activated protein kinase-kinase (MEK) resulting in the activation of ERK/MAPK [223]. Activated ERK/MAPK can then activate other protein kinases or migrate to the nucleus to activate transcription factors, such as c-JUN, c-MYC and c-FOS, important in the cell cycle [225].

However, the RAS/MAPK pathway can also be activated independently of IRS via direct binding of Grb2 to the receptor's SH2 domain [222, 223].

**Janus Kinase/Signal Transducers and Activators of Transcription Pathway**

**Figure 3:**
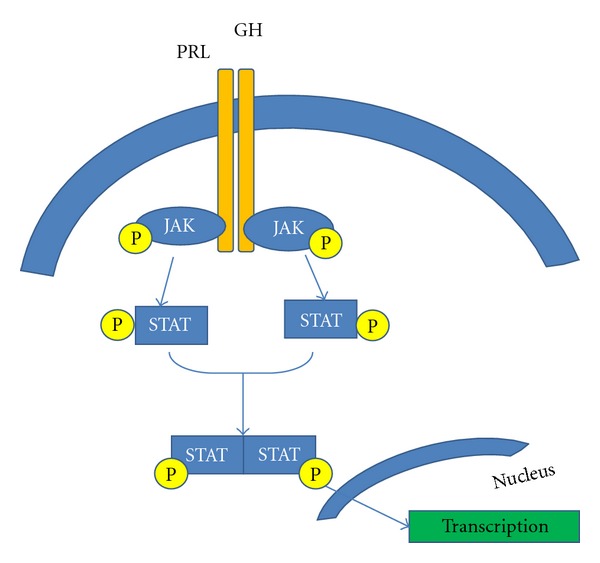
Schematic representation of JAK/STAT signalling. Mechanisms through prolactin and growth hormone modulate beta-cell proliferation. These hormones via prolactin receptor activate JAK, which is able to phosphorylate STAT. After phosphorylation, two molecules of STAT bind together and translocate into the nuclei where elicit their transcriptional function.

Another important pathway in beta-cell biology is the JAK/STAT pathway. Both growth hormone (GH) and prolactin have been shown to mediate their effects via this pathway [226, 227]. Once activated, Janus kinases (JAKs) are able to phosphorylate signal transducers and activators of transcriptions (STATs) which enter the nucleus to regulate transcription of target genes. In particular, JAK2 and STAT-5 have been shown to regulate beta-cell growth and survival [226–228], as well as to reduce apoptosis [229]. More recently, it was recognized that the JAK/STAT pathway is regulated by Suppressors of cytokine signaling (SOCS). These suppressor proteins inhibit STAT phosphorylation by binding to phosphorylated JAKs, or compete with STATs for phosphotyrosine binding sites on cytokine receptors. Furthermore, SOCs facilitate the degradation of JAKs [230] (Figure 3). Its effects have resulted in reduced beta-cell proliferation in a sex-dependent manner [231].

**c-*Myc***

The cellular proto-oncogene, c-*Myc*, encodes the protein c-Myc whose key biological function is to promote cell cycle progression. However, this enigmatic protein appears to be a key player in various other biological processes, such as differentiation, cell death, and angiogenesis.

Recent studies by several laboratories, including our own, support the notion that c-Myc may have a central role in the regulation of beta-cell mass required for replication (during the G_1_/S transition), cell growth, and apoptosis [227].

It is likely that blood glucose levels influence the regulation and expression of c-Myc in beta-cells. In fact, it was observed that expression of c-Myc was stimulated by high glucose in a Ca^2+^-dependent manner and also by cAMP [232]. High glucose also increases c-Myc mRNA levels in rat islets *in vitro* [233]. Therefore, taken together, these studies support the opinion that high blood glucose levels may activate beta-cell replication through c-Myc expression.

Besides, the actual mechanism by which hyperglycaemia activates c-Myc is fully clear. It is known that cell signalling of c-Myc pass through cyclin E-CDK2 activity early in the G_1_ phase of the cell cycle, an essential event in c-Myc-induced G_1_-S progression [234, 235].

In particular, it is well known that CCND2 (which encodes cyclin D2) and CDK4 are direct target genes of c-MYC [236, 237]. Expression of CCND2 and CDK4 leads to sequestration of p27, the CDK inhibitor, in cyclin D2–CDK4 complexes [236]. The subsequent degradation of p27 has been shown to involve two other c-MYC target genes, CUL-1 and CKS [237]. By preventing the binding of p27 to cyclin E-CDK2 complexes, c-MYC allows inhibitor-free cyclin E-CDK2 complexes to become accessible to phosphorylation by cyclin activating kinase (CAK). Increased CDK2 and CDK4 activities would result in Rb hyperphosphorylation and subsequent release of E2F from Rb.

However, the final result of c-Myc activation on beta-cell is peculiar and differs in various tissues. For example, in contrast of skin, the predominant effect of activating c-MYC in pancreatic beta-cells of adult transgenic mice (cMYCER^TAM^) is apoptosis and not proliferation, resulting in development of diabetes [46, 238].

However, concerning cell signalling mechanism involved in cMYC-induced apoptosis, D cyclins do not appear to be required for c-MYC-induced apoptosis *in vitro* [239], indicating that these two major functions of c-MYC (proliferation and apoptosis) may involve, at least in part, distinct sets of downstream mediators.

**Calcineurin/Nuclear Factor of Activated T-Cells**

More recently a new important pathway able to regulate beta-cell mass and function has been identified. The key components of this pathway are Ca^2+^/calmodulin-dependent protein phosphatase 2-b (or calcineurin-b) and nuclear factor of activated T cells (NFAT).

In beta-cells calcineurin is activated by an increase of cytosolic Ca^2+^, once activated, it can dephosphorylate NFAT transcription factors, which translocate into the nucleus where bind to consensus NFAT binding elements on specific gene promoters resulting in the activation of gene transcription [240]. On the contrary, some specific NFAT kinase can phosphorylate NFAT proteins and then inactivate and exporting them from nucleus.

In particular, it was reported in transgenic NFAT activated mice that NFAT in beta-cells can induce proliferation activating transcription of genes coding for cyclin D1, cyclin D2, and CDK4 [159].

More recently, another possible way to regulate the beta-cell mass has been reported. In fact, it was observed that calcineurin/NFAT pathway is implicated in the glucose regulation of IRS-2 gene expression. In particular, NFAT activated can bind to IRS-2 promoter enhancing its transcription and it was prevented by specific inhibition of NFAT [241]. This positive effect on beta-cell survival observed was also previously described [242]. Unfortunately, until now, the mechanisms involved in the regulation of beta-cell cycle by calcineurin are partially understood.

The important role of calcineurin/NFAT on beta-cell proliferation was highlighted using different transgenic models. Using mice with beta-cell-specific deletion of the Calcineurin phosphatase regulatory subunit, Calcineurin b1 (Cnb1), has shown a reduction of beta-cell function, proliferation and mass, and it coincided with reduced expression of normal regulators of beta-cell proliferation. Similarly, long-term activation of calcineurin induces impaired glucose tolerance by alterations in beta-cell mass, and the activation of calcineurin signalling negatively affects proliferation and survival of beta-cells [243].

On the contrary, in normal adult beta-cells, conditional NFAT activation has shown to promote expression of cell-cycle regulators resulting in an increase of beta-cell proliferation and mass, resulting in hyperinsulinaemia [159].

### 2.3. Cell Cycle/Molecular Pathways of Beta-Cell Proliferation

The critical point in the beta-cell cycle seems to be the G_1_S “checkpoint.” At this point, the cell is committed to DNA replication, and it is regulated by the retinoblastoma protein (Rb). Rb is critical because it has the ability to stall the cell cycle. Rb itself is inactivated by a group of serine threonine protein kinases, the cyclin-dependant kinases (CDKs) including CDK4/6 and CDK2, which in turn are negatively regulated by cyclin-dependent kinase inhibitors (CDKIs).

- *Rb* is a member of a family of pocket proteins which is able to block G_1_S transition by binding to E2F transcriptional activator proteins. When liberated from Rb, E2F transcription factor elicits its activity in controlling the transition from G_1_ to S phase.
- Mice deleted for *E2F-1* display defective pancreatic growth and islet dysfunction [187]. In *E2F-1* and *E2F-2* double knockout mice, both exocrine and endocrine glands had atrophied by 3 months of age, a feature not seen in single knockouts at the same time point. Diabetes subsequently followed, along with an increased rate of apoptosis [182].
- The *cyclin-dependent kinase* CDK4 and 6 are activated by Cyclin D (in particular, D1 and D2) whereas CDK2 is activated by Cyclin E. Both Cyclins D1 and D2 are essential for normal postnatal islet growth. In adult islets, global deletion of cyclin D2 fails to stimulate adequate compensatory upregulation of cyclin D1 within islets and drastically damages postnatal beta-cell proliferation, islet mass, and decreases glucose tolerance [7, 189]. The importance of cyclin D1 has been highlighted using transgenic mice that overexpress cyclin D1. In this animal model, an increase in both beta-cell mass and proliferation was shown [190].
- CDK4 has also been shown to be a key regulator of beta-cell cycle [244]. Knockout studies have shown a very specific phenotype, with beta-cell hypoplasia and severe diabetes [191, 194, 245], whilst studies using an activating mutation in CDK4, rendering it resistant to p16, have resulted in pancreatic hyperplasia, which demonstrated normal physiology [191, 192]. Other studies have shown up to a three-fold increase in beta-cell proliferation with CDK4 overexpression [191, 193].
- The CDKs are inhibited by two groups of CDK inhibitors (CDKIs) which are also expressed in islets, *INK4 proteins* (p16, p15, p18, p19 inhibit CDK 4 and CDK6), and *CIP/KIP proteins* (p21, p27 and p57). Whilst INK4 family proteins promote cell cycle arrest, p21 and p27 are integrated into the cyclin D/Cdk4 complex that is able to arrest cell cycle progression through dephosphorylation of Rb protein [246], on the other hand, they are able to inhibit cyclin E/A CDK2 complex resulting in a block of phosphorylation of Rb protein [247].
- Various studies have looked at the effects of CDKIs on islets. In p21^−/−^ mice, islets were phenotypically and metabolically comparable to their wildtype counterparts, possibly due to upregulation of p57, whereas loss of function of p57 has been associated with hyperinsulinaemia at infancy [248]. Actively inhibiting the effect of p18, using knockout mice resulted in beta-cell hyperplasia of up to 40% that appears to be CDK4 dependent [245].
- In contrast, p27 has been shown to partially reverse the islet phenotype independent of CDK4. Overexpression of p27 in beta-cells of transgenic mice impairs beta-cell proliferation, resulting in decreased beta-cell mass and diabetes [195]. Furthermore, p27^−/−^ mice increases beta-cell proliferation doubling their beta-cell mass at birth, and this expansion was accompanied by increased insulin secretion [249].

## 3. Role of MicroRNAs on the Beta-Cell Mass Regulation

MicroRNAs (miRNAs) are small 19–23 nucleotide noncoding RNA molecules that act as posttranscriptional regulators of different genes involved in various cellular processes, such as apoptosis, differentiation, and proliferation. This regulation pass through Generally, miRNAs can regulate protein synthesis either by repressing translation and/or by degradation through deadenylation of mRNA targets [250, 251].

The role of miRNAs in beta-cell mass regulation is not yet fully understood. The most studied miRNA in this contest is miR-375. It was reported that its overexpression attenuates proliferation of beta-cells and glucose-induced insulin secretion [16–18]. Indeed, ectopic expression of miR-375 in diabetic pancreatic beta-cells results in increased susceptibility to fatty acid induced apoptosis [16]. Moreover, it was reported that in ob/ob mice in which miR-375 was deleted, they develop a marked decrease in beta-cell mass, which results in a severe insulin-deficient diabetes not found in ob/ob mice [15]. Therefore, it is becoming clear that miR-375 targets a suit of genes that negatively regulate cell growth and proliferation and that aberrant loss of this miRNA leads to dramatic reduction of beta-cell mass [15].

Furthermore, it was reported that overexpressions of both miR-34a and miR-146a are involved in programmed cell death, in particular, in apoptotic processes. In contrast, reducing miR-34a or miR-146a levels did not affect cell survival.

In fact, prolonged beta-cell exposure to palmitate (FFA) and proinflammatory cytokines changes/increases the expression of miR-34a and miR-146a, and treatment with anti-miR34a or anti-miR148 diminished the number of death cells in the presence of apoptotic stimulus.

The effect on the regulation of apoptosis may be due to the capacity of miR-34 to control the expression of the antiapoptotic protein Bcl2 [252], meanwhile miR-146a may control the apoptotic process through the regulation of the NF-kB pathway [253].

## 4. Beta-Cell Mass Plasticity

In addition to maintaining beta-cell mass under normal circumstances, as just discussed, an organism must also be able to alter its beta-cell mass in accordance with insulin's requirements. In particular conditions, such as pregnancy and obesity, beta-cell mass enlargement is observed [2]. However, when compensatory beta-cell mass expansion is inadequate, gestational diabetes in the case of pregnancy and T2D in the case of obesity are the results.

Although the majority of humans do not become diabetic in these circumstances, a significant part of the population is predisposed to beta-cell failure, for currently unknown reasons. The precise mechanism of beta-cell mass maintenance and expansion, that is, proliferation, neogenesis, or increase in size, has been elucidated only in part [178, 254, 255].

### 4.1. Reversible Beta-Cell Mass Expansion during Pregnancy

Different studies in rodents found a two- to five-fold increase in beta-cell mass during gestation [255, 256] and demonstrated an involvement of both beta-cell hypertrophy [178, 256, 257] and proliferation [256, 257]. Van Assche et al. [254] have reported that in humans during pregnancy the beta-cell mass increased 2.4-fold compared with nonpregnant women. Unfortunately, human data are limited to few studies using a small number of subjects. Butler et al. [258] have reported an approximately 1.4-fold increase in beta-cell mass using 18 women. In contrast to rodents, beta-cell mass expansion in humans was achieved by the formation of new islets or islet neogenesis [258].

The main stimuli for beta-cell proliferation during pregnancy are placental lactogen (PL), prolactin (PRL), growth hormone (GH) [178, 259], and serotonin [260]. Postpartum, in rodents, beta-cell mass returns to normal levels within 10 days through increased beta-cell apoptosis, decreased beta-cell proliferation, and reduced beta-cell size [255].

**Placental Lactogen**

Placental lactogen (PL) hormone has been implicated as the primary factor responsible for the enhanced islet mass and function that occur during pregnancy [178, 259]. PL interacts with receptors in the PRL/GH receptor family, stimulating the JAK-2/STAT-5 intracellular signalling pathway [261, 262].

Prior studies performed *in vitro *and over the short term suggested that PL is a more powerful islet mitogen than GH or PRL [178, 263]. These data were confirmed in *in vivo* studies using transgenic mice expressing mouse PL-1 under the control of the rat insulin promoter (RIP-mPL1) [259, 264]. The expansion of beta-cell mass in RIP-mPL1 was attributed principally to a two-fold increase in beta-cell proliferation and a 20% increase in beta-cell size (hypertrophy). Interestingly, islet number was not significantly increased when compared to wild-type mice [259]. These findings were supported by a PL receptor knockout study, which showed a significant reduction in beta-cell mass [265]. Therefore, PL could offer a novel therapeutic target to treat diabetes.

**Prolactin**

Prolactin (PRL), a hormone, acts through its specific receptor, prolactin-receptor (PRLR) to induce beta-cell proliferation *in vitro* [263]. Targeted deletion of the PRLR reduces beta-cell mass and mildly impairs insulin secretion [265]. The pivotal role of the PRLR for beta-cell adaptation during pregnancy was demonstrated using pregnant mice heterozygous for the PRLR null mutant. These mice exhibited reduced beta-cell proliferation, and impaired glucose tolerance [266]. It has been extensively demonstrated in different *in vitro* studies that PRL binding to PRLR is able to activate different signaling pathways such as STAT5, MAPK, and IRS/PI3K resulting in enhancing beta-cell mass during pregnancy [267–269]. It is currently unclear whether PRL and/or PL stimulate these pathways *in vivo*.

**Menin**

More recently, menin (a tumour suppressor encoded by the *Men1* gene) has been linked to the regulation of beta-cell proliferation during pregnancy [157]. Expression of menin, p18, and p27 are reduced during pregnancy in maternal islets, thus leading to islet mass expansion to meet the increased metabolic demand. After birth, menin returns to normal. Interestingly, menin is regulated by prolactin [157], supporting menin as an important mediator of beta-cell proliferation during pregnancy. It has also been proposed that, during pregnancy, activation of JAK2 and AKT, in response to prolactin, leads to increased p21 expression, whereas menin and p18 expression are suppressed. It is well known that AKT and p21 induce beta-cell proliferation, meanwhile the downregulation of menin and p18 allows for enhanced beta-cell proliferation [270].

**Serotonin**

A recent study by Kim et al. [260] shows that beta-cells, like serotoninergic neurons, are able to synthesize, store, and secrete serotonin (5-HT) as well as express the specific serotonin receptors (5-HTR2B and 5-HTR1D). These authors reported that, in pregnant mice, 5-HTR2Bexpression increased significantly during midgestation (day 6 to 16) and normalized at the end of gestation, whereas 5-HTR1D expression increased at the end of gestation (day 17) and postpartum. Notably, increased 5-HTR2Bexpression closely correlated with the period of increased beta-cell proliferation, and increased 5-HTR1Dexpression correlated with the cessation of beta-cell proliferation and regression of beta-cell mass. Moreover, it was reported that in pregnant mice stimulation with high concentrations of prolactin induces a strong increase in the serotonin expression compared with nonpregnant control mice.

These results suggest that during pregnancy lactogenic signalling induces serotonin expression and synthesis in islets able to stimulate beta-cell proliferation through the 5-HTR2B pathway. Shortly before parturition, expression of 5-HTR2B decreases and 5-HTR1D expression increases, resulting in the reduction of beta-cell proliferation and beta-cell mass. In this way, beta-cell mass returns rapidly to prepregnancy levels.

### 4.2. Compensation for Body Mass

Excessive increase in body weight leads to obesity. Obesity is linked/correlated with insulin resistance and associated with compensatory physiological response at the level of beta-cell mass increase. As previously described, when compensatory beta-cell mass expansion is inadequate, T2D in the case of obesity is consequential.

Diet-induced obesity results in insulin resistance and beta-cell mass expansion in humans and mice. In nondiabetic animal models of obesity, for example, the C57Bl/6 mouse strain which is notoriously susceptible to these effects, there is a 2.2-fold increase in beta-cell mass and proliferation after 4 months of a high-fat diet versus a control diet [271]. Meanwhile, in the Zucker diabetic fatty (ZDF) *fa/fa* rat, beta-cell mass increased four folds compared with lean controls [79]. There is also evidence for increased beta-cell mass in nondiabetic obese humans [51].

However, these mice eventually become diabetic and lose their beta-cell mass due to increased beta-cell apoptosis and reduced beta-cell proliferation.

In genetic models of obesity and insulin resistance, there is a compensatory expansion of beta-cell mass. For example, in mice lacking a functional leptin receptor (db/db mice), beta-cell mass exhibits two-fold beta-cell mass increase [115]. The Zucker rat (*fa/fa*) also has a homozygous mutation in the gene encoding the leptin receptor. ZDF rats exhibit increased beta-cell mass and increased beta-cell proliferation prior to the onset of diabetes, but increased beta-cell apoptosis prevents them from adequately expanding their beta-cell mass after the onset of diabetes, despite continued high rates of beta-cell proliferation [79]. This phenotype contrasts with that observed in nondiabetic Zucker fatty (ZF) rats, which possess the same mutation as ZDF rats and also become obese and insulin resistant but do not develop diabetes due to sufficient beta-cell mass expansion through increased beta-cell proliferation, neogenesis, and hypertrophy [79].

## 5. Conclusions

On the basis of available research findings that we have discussed above, we can propose that (i) postnatal beta-cell mass sizes in response to changing metabolic demands, (ii) is carried out by an interaction of beta-cell replication (proliferation and/or neogenesis) and apoptosis, and (iii) this process is regulated by different growth factors/nutrients that interact between them.

Actually, our present knowledge to the understanding of proliferation, neogenesis, and apoptosis is still incomplete.

The balance between apoptosis and replication seems to be pivotal in a right beta-cell mass maintenance. Failure in this balance results in beta-cell dysfunction and consequently diabetes onset. Because of the worldwide diffusion of this pathology, new therapies able to restore the original beta-cell mass and its functionality are under continuous development. Islet transplantation is already under clinical investigation but for the foreseeable future it will be very restricted in application due to the limited supplies of human cadaveric islets for transplantation. As a result, there is intense interest in developing other sources of new beta-cells and identification of new molecules able to restore beta-cell mass in diabetics.

## Figures and Tables

**Table 1:** Hormones, growth factors, and other factors regulating beta-cell mass.

	Inhibitors	Stimulators
Metabolites	Glucose, FFA	Glucose, FFA, amino acids
Cytokines	IL-1, IFN-g, TNF-a, leptin	GH, PRL, PL
Growth factor family	HGF	IGF-I, IGF-II, insulin, TGF-a, betacellulin, HB-EGF, aFGF, VEGF, PDGF, HGF
Placental hormones		placental lactogen, prolactin
Glucagon family		GLP-1, GIP, glucagon
Somatostatin family	Somatostatin	
CGRP family	IAPP/amylin	
Gastrin family		Gastrin, CCK
TGF-b family	TGF-b, follistatin	Activin A
Neurotrophins		NGF, NT-3
Neurotransmitters	(Nor)epinephrine	Acetylcholine
Lectins		Reg/INGAP/PSP/PTP
Adhesion molecules		Integrin a6b1, Cx43, Cx36
Drugs	Diazoxide	Nicotinamide, SU
Toxins	Streptozotocin, alloxan	

Modified from Nielsen et al. [29]. aFGF, acidic fibroblast growth factor; CCK, cholecystokinin; Cx. Connexin; HB-EGF, heparin-binding EGF-like protein; HGF, hepatocyte growth factor/scatter factor; IAPP, islet amyloid polypeptide; IFN, interferon; IL, interleukin; INGAP, islet neogenesis- associated peptide; NGF, nerve growth factor; NT-3, neurotrophin-3; PDGF, platelet-derived growth factor; PSP, pancreatic stone protein; PTP, pancreatic thread protein; SU, sulfonylurea; TGF, transforming growth factor; TNF, tumor necrosis factor; VEGF, vascular endothelial growth factor.
